# The metazoan parasite communities of the shoal flounder (*Syacium gunteri*) as bioindicators of chemical contamination in the southern Gulf of Mexico

**DOI:** 10.1186/s13071-014-0541-3

**Published:** 2014-11-27

**Authors:** Víctor Manuel Vidal-Martínez, Oscar A Centeno-Chalé, Edgar Torres-Irineo, Juan Sánchez-Ávila, Gerardo Gold-Bouchot, M Leopoldina Aguirre-Macedo

**Affiliations:** Laboratorios de Parasitología y Geoquímica, Centro de Investigación y de Estudios Avanzados del Instituto Politécnico Nacional, Unidad Mérida, Km 6 Carretera Antigua a Progreso, Cordemex, Mérida, Yucatán 97310 México

**Keywords:** Parasite communities, Bioindicators, Environmental impact, Flatfish, Contamination, Gulf of Mexico

## Abstract

**Background:**

Because agriculture and offshore oil extraction are significant economic activities in the southern Gulf of Mexico, high concentrations of nutrients and hydrocarbons are expected. As parasite communities are sensitive to environmental impacts, these contaminants should have an effect on metrics such as species richness, relative abundance and similarity. Consequently, these community metrics can be used as indicators of aquatic environmental health. Our objectives were to describe the parasite communities of the shoal flounder *Syacium gunteri* and to determine potential thresholds above which environmental contaminants become major controlling factors of parasite community metrics.

**Methods:**

The study area included 33 sampling sites in the southern Gulf of Mexico, where benthic sediments, water and shoal flounder individuals were collected. Data on ecto- and endo-parasites from flounder and nutrients, contaminants and physicochemical variables from the water and sediments were obtained. The statistical associations of the parasite community metrics at the component and infracommunity levels and the environmental data were analysed using redundancy analysis (RDA).

**Results:**

Overall, 203 shoal flounder were examined for parasites, recovering 13 metazoan parasite species, and 48 physicochemical (e.g. temperature, nutrients) and contaminant (e.g. hydrocarbons, heavy metals) variables were obtained. The larval stages of the cestode *Oncomegas wageneri* and the nematodes *Pseudoterranova decipiens* and *Hysterothylacium* sp. were numerically dominant at the component and infracommunity levels. The parasite community metrics had significant negative statistical associations with both nitrate and total PAHs. With the exception of these two chemicals, which exceeded the threshold effect levels (TELs), no other environmental variable exceeded the range considered safe for marine organisms.

**Conclusions:**

The community metrics chosen generally had robust statistically significant associations with both physicochemical and contaminant variables, which supports the ecological relevance of these parameters as indicators of aquatic environmental health. Within the study area, the shoal flounder and their parasites live in a polluted environment with relatively high levels of hydrocarbons and nitrate. Regarding nitrate, we emphasise that if uncontrolled sewage discharge continues in the southern Gulf of Mexico, hypoxic conditions similar to those caused by the Mississippi river can be expected in the near future.

**Electronic supplementary material:**

The online version of this article (doi:10.1186/s13071-014-0541-3) contains supplementary material, which is available to authorized users.

## Background

Marine biologists currently recognise that both marine free-living benthic organisms and their parasites are useful as bioindicators of aquatic environmental health, i.e., as species or communities used to assess the quality of the environment and how it changes over time [1-6]. Parasites of aquatic organisms are good bioindicators of environmental impact because their populations and communities are sensitive to environmental insults [5,7-9]. For example, parasite abundance in Finnish lakes that were highly polluted with pulp and paper effluents (PPE) was lower than in lakes with lower PPE levels [10]. After environmental conditions in the lakes improved, parasites returned to levels similar to those in less polluted lakes. At the community level, Huspeni and Lafferty [11] used larval digenean infections of snails as bioindicators of environmental recovery in salt marshes. After six years, the number of infected snails in the impacted zones recovered to at least as high as in the control zones. Recently, two meta-analyses have noted the usefulness of parasite communities as a bioindicator of the recovery of impacted habitats [5,12]. Vidal-Martínez [5] showed that parasite communities have a positive interaction term with eutrophication, meaning that this environmental impact increases several community metrics such as species richness or relative abundance.

To monitor the effect of the contamination in the southern Gulf of Mexico produced by the oil industry and other environmental insults such as unregulated sewage discharge or pesticide transport by river run-off, studies have been performed on species of parasitic helminths and protozoans of the Mayan catfish *Hexanematichthys assimilis* [13], the pink shrimp *Farfantepenaeus duorarum* [14], and the pufferfish *Spheroides testudineus* [15]. These studies have concluded that chemical contamination influences parasite infection parameters.

During studies in the southern Gulf of Mexico to determine the environmental quality of sediments and water for the Mexican Petroleum company (PEMEX), we obtained data on the helminth parasites infecting the shoal flounder *Syacium gunteri*. Given that agriculture and offshore petroleum extraction are significant economic activities in the southern Gulf of Mexico [16], high concentrations of nutrients from river run-off, hydrocarbons, and other contaminants such as heavy metals or pesticides would be expected. Because parasite communities are sensitive to environmental impacts, we hypothesise that these contaminants should have a direct effect on parasite community metrics such as species richness, relative abundance or similarity within and among sampling sites. As the parasite communities of *S. gunteri* have yet to be described for the region, the objectives of this study were twofold: 1) to describe the parasite communities of the shoal flounder at the component and infracommunity levels and 2) to determine whether there are thresholds above which environmental contaminant levels become major controlling factors of parasite community metrics.

## Methods

### Study area and sampling procedures for sediments

**Figure 1:**
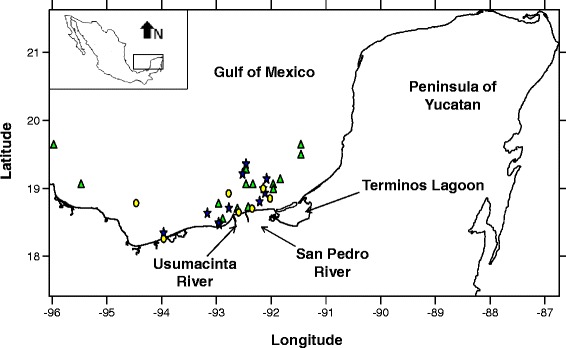
**Sampling sites in Campeche sound, Gulf of Mexico, where sediment and water samples were obtained.** The shoal flounders *Syacium gunteri* were caught in trawls around each station. Symbols were as follows: sites of group 1; sites of group 2; sites of group 3.

The study area included 33 sampling stations in the southern Gulf of Mexico (Figure 1). Benthic sediments were collected between September 6 and October 8, 2005, at depths between 1.30 and 253.5 m from the oceanographic vessel (OV) Justo Sierra using a 0.25 m^2^ Hessler Sandia MK-III box corer. Water samples were taken at 5 m depth intervals using 1-gallon amber glass bottles, which were closed under water to avoid contamination with surface mixtures. We obtained 48 physicochemical parameters from water and sediments including oxygen (mg/L), salinity (UPS), pH, and nitrogen concentrations (see Additional file 1 of the online supporting material for a complete list per sampling site). Sediment samples were placed in high-density polythene (HDPE) bags and maintained at 4 °C for transport to CINVESTAV-IPN, Unidad Mérida. Hydrocarbon sampling procedures have been described elsewhere [14,17,18]. The physicochemical characteristics, hydrocarbon and metal concentrations of the sediment were determined in the Marine Chemistry and Geochemistry laboratories, respectively, by applying standardised methods [19-21]. Principal coordinates of neighbour matrices (PCNM) analyses were used to generate a set of spatial variables from the geographical position of each station [22,23]. These spatial variables (called PCNM vectors) are spectral decompositions of the spatial relationships among stations corresponding to all the spatial scales that can be perceived from the data [23]. To calculate these variables, we followed the procedure of Santana-Piñeros [24]. Both environmental and biological data were obtained within a very narrow window of time (one month). Therefore, the temporal variability in both types of data is expected to be minimal, and thus, most of the variability in the data should be spatial.

### Sampling procedures for flatfishes and helminth parasites

Fish were collected using 1 h trawls with 20 m shrimp nets. The trawls lasted 50–60 minutes at 0.6-0.7 knots around each station. The shoal flounder *Syacium gunteri* were kept in isolated plastic bags in coolers in the cold storage of the vessel and transported to CINVESTAV-IPN Unidad Mérida for parasitological examination. The total length, standard length, maximum height (cm) and weight (g) were recorded for each individual fish. Subsequently, the body surface, cavities and all internal organs were individually examined using a dissection microscope. The metazoan parasites were counted in situ and preserved in 70% alcohol (digeneans) or 4% formalin (nematodes) in labelled vials for subsequent taxonomic identification. Digeneans, cestodes and acanthocephalans were stained using the Mayer paracarmine technique, and the nematodes were cleared using increasing concentrations of glycerine [25]. Voucher specimens were deposited in the National Collections, Universidad Nacional Autónoma de México (CNHE).

Prevalence and mean abundance were calculated for each metazoan parasite species following Bush et al. [26]. Host-specialist helminth species were those previously reported from fish species of the same genus or fish family, whereas generalist species were those previously reported in fish species from different fish families. Species richness and similarity were calculated separately at the component and infracommunity level [27] for total communities (i.e., including every possible infected organ). Autogenic species were those completing their life cycles within aquatic environments. In contrast, allogenic species were those reaching sexual maturity in piscivorous birds or terrestrial mammals [28]. An infracommunity was defined as all of the metazoan parasites infecting an individual fish. At the infracommunity level, the mean ± SD (standard deviation) of the number of species and individual helminths per host examined were calculated. Brillouin’s diversity index [29] was calculated for all infracommunities and expressed as the mean ± SD for each host sample. The Berger-Parker dominance index [29] was calculated at the infracommunity level for the parasites of each individual shoal flounder and presented as the mean ± SD of the index per sampling site. The component community included all of the metazoan parasites infecting a sample of flatfishes of the same species at a specific sampling site. At the component community level, the total number of species and the total number of individual metazoan parasites per sampling site were obtained. The Berger-Parker dominance index [29] was calculated at the component community level for the parasite fauna of the sample of shoal flounder at each sampling site. The qualitative and quantitative faunal similarities were determined using Jaccard’s similarity index and the percentage of similarity [29] for all possible combinations within and among sampling sites at both the component and infracommunity levels. Data normality was determined by using rankit plots [30]. Variables with large deviations from normality were natural log-transformed. One-way ANOVA was used to determine potential differences among the mean numbers of species and individuals per sampling site. Where transformation did not improve normality, the non-parametric Kruskal-Wallis (KW) test was used. The significance of all statistical analyses was established at α <0.05, unless otherwise stated. We focused on community metrics to obtain a more holistic view of the response of parasites rather than concentrating on individual parasite species. Due to the large number of sampling sites, we examined whether these sites would group based on the parasite species composition. Thus, we used cluster analysis with UPGMA (Unweighted Pair Group Method with Arithmetic Mean) as a hierarchical clustering method and Jaccard’s similarity index at the component community level to determine the possible presence of groups of sampling sites that were similar due to their parasite species composition. The cluster analysis was undertaken using MVSP V. 3.22. Then, the tree obtained was then arbitrarily cut at 0.5 of Jaccard’s similarity. Thus, the multivariate analysis (see below) was applied to the original 33 sampling sites and to the groups of sampling sites produced by the cluster analysis.

By using detrended correspondence analysis (DCA), the length of the ordination axes scale (gradient) in standard deviation units (SDU) for the species data was found to be 0.06 SDU. This SDU value indicates that our data only include a limited portion of the environmental range of the shoal flounder, and this analysis assumes linear associations between the environmental and biological variables. Because the SDU value was less than 3, the recommended option for analysis is redundancy analysis (RDA) [31]. RDA is a constrained form of both PCA and multivariate multiple regression and was applied using CANOCO [31,32] to determine possible associations between environmental variables and the infracommunity and component community metrics. Covariable analysis was used to control for confounding variables such as standard length and weight and to determine the actual influence of independent environmental variables on the infracommunity and component community metrics. Monte Carlo tests were used to determine the significance of the canonical axes for both parasite community metrics and environmental variables. The use of constrained models decreased the percentage of explained variance, although the models were still statistically significant [31].

## Results

**Figure 2:**
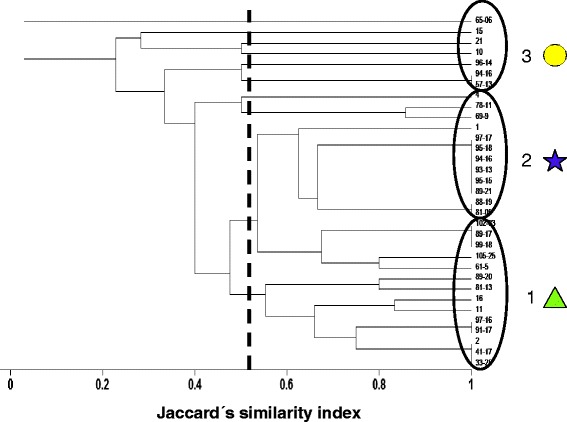
**Groupings based on the parasite species of** ***Syacium gunteri*** **shared among sampling sites.** The dendogram was produced using the unweighted pair-group method (UPGMA) and Jaccard’s similarity index. Note that the symbols for groups 1 (), 2 (), and 3 () are the same as in Figure 1.

A total of 203 shoal flounder from 33 sampling sites (Figure 1) were examined for parasites, and 13 metazoan parasite species were recovered. The cluster analysis showed the presence of three groups of sampling sites, called groups 1, 2 and 3 (Figure 2). There were no significant differences in fish standard length (ANOVA ONE-WAY, F_[2, 200]_ = 1.61, p = 0.20) or weight (ANOVA ONE-WAY, F_[2, 200]_ = 3.09, p = 0.05) among the three groups. In contrast, Fulton’s condition factor for the fish from group 1 was significantly higher than for groups 2 and 3 (ANOVA ONE-WAY, F_[2, 200]_ = 4.36, p =0.01, n = 203). The metazoan parasites infecting the shoal flounder were two digeneans (one larval and one adult), five nematodes (four larvae and one adult), four cestodes (all in the larval stage), one adult acanthocephalan and one adult parasitic copepod (Table 1). The prevalence and mean abundance of the metazoan parasites infecting the shoal flounder are summarised in Table 1 for all 33 sampling stations in the overall category and for groups 1 through 3. In all of the groups of sampling sites, the most frequent and abundant species was the larval cestode *Oncomegas wageneri*, followed by the larval nematodes *Pseudoterranova decipiens* and *Hysterothylacium* sp. Overall, there was no significant difference among groups (ANOVA ONE-WAY, F_[2, 200]_ = 3.06, p = 0.05) in the mean abundance of *O. wageneri*. However, there were significant differences in the mean abundance of *P. decipiens* among the groups (ANOVA ONE-WAY, F_[2, 200]_ = 4.85, p = 0.008), with Tukey’s test indicating that group 2 had significantly more *P. decipiens* than did groups 1 and 3 (p <0.05). The remainder of the metazoan parasites in Table 1 had a low prevalence and low mean abundance, which in turn precluded meaningful statistical analysis.

**Table 1:** **The metazoan parasites of the shoal flounder** ***Syacium gunteri*** **in the southern Gulf of Mexico**

		**Overall**		**Group 1**		**Group 2**		**Group 3**	
Number of fish examined		203		102		46		55	
Standard length (cm)		11.94 ± 3.53		11.65 ± 3.31		12.79 ± 4.26		11.92 ± 3.24	
Weight (g)		37.59 ± 45.35		33.24 ± 43.02		44.29 ± 43.97		40.42 ± 50.52	
Body condition (Fulton’s index)		0.018 ± 0.005		0.018 ± 0.005		0.018 ± 0.003		0.020 ± 0.003	
	**CN**	**%**	**MA ± SD**	**%**	**MA ± SD**	**%**	**MA ± SD**	**%**	**MA ± SD**
**Digenea**									
*Lecitochirium* sp.^A,I^	9341	2.00	0.04 ± 0.31	-	-	9.30	0.16 ± 0.65	-	-
*Stephanostomum* sp.^L,F^	9342	17.50	1.25 ± 5.36	18.63	1.28 ± 6.06	37.21	2.74 ± 6.59	-	-
**Nematoda**									
*Anisakis typica* ^L,Me^	9343	0.50	0.01 ± 0.07	-	-	2.33	0.02 ± 0.15	-	-
Capillariidae gen. sp.^A,I^	9344	1.50	0.03 ± 0.26	2.94	0.06 ± 0.37	-	-	-	-
*Hysterothylacium* sp*.* ^L,Me^	9345	17.00	1.58 ± 4.48	13.73	0.93 ± 2.84	39.53	4.56 ± 7.66	5.45	0.44 ± 2.03
*Pseudoterranova decipiens* ^L,Me^	9346	53.00	3.10 ± 5.27	53.92	2.69 ± 4.34	51.16	5.23 ± 7.58	50.91	2.18 ± 4.21
*Spirocamallanus halitropus* ^A,I^	9347	3.50	0.09 ± 0.62	0.98	0.01 ± 0.10	9.30	0.28 ± 1.16	3.64	0.09 ± 0.55
**Cestoda**									
*Nybelinia* sp.^L,I^	9348	2.50	0.23 ± 1.94	0.98	0.01 ± 0.10	9.30	1.05 ± 4.11	-	-
*Kotorella pronosoma*.^L,I^	9349	4.00	1.47 ± 8.55	4.90	2.02 ± 10.09	6.98	2.02 ± 9.88	-	-
*Oncomegas wageneri* ^L,I^	9350	81.50	35.72 ± 45.49	83.33	37.83 ± 50.62	76.74	24.19 ± 38.33	81.82	40.80 ± 39.38
Tetraphyllidea gen. sp.^L,I^	9351	0.50	0.09 ± 1.27	-	-	2.33	0.42 ± 2.74	-	-
**Acanthocephala**									
*Serrasentis sagittifer* ^A,I^	9352	4.50	0.12 ± 0.79	5.88	0.14 ± 0.76	6.98	0.23 ± 1.23	-	-
**Arthropoda**									
*Caligus pomacentrus* ^A,G^	29653*	2.00	0.02 ± 0.14	0.98	0.01 ± 0.10	6.98	0.07 ± 0.26	-	-

The infection parameters are prevalence (%) and mean abundance (MA ± standard deviation (SD)) for 33 sampling sites l (Overall) and the three groups of sampling sites formed by cluster analysis (Figure 2). ^A^Adult parasite, ^F^fins, ^G^gills, ^I^intestine, ^Me^mesenteries, ^Mu^muscle, ^L^larval parasite, CN = Catalogue number of the National Helminthological Collection, Universidad Nacional Autónoma de México, *National Crustacean Collection (CNCR), Universidad Nacional Autónoma de México.

### Component communities

The metazoan parasite fauna of the shoal flounder consisted of 13 species, with groups 1 and 2 containing 12 and 10 species, respectively, and group 3 containing the lowest number of species (4) (Table 2). There were significant differences in the total number of species per sampling site among groups (ANOVA ONE-WAY, F_[2, 30]_ = 3.76, p = 0.03), with Tukey’s test indicating that group 3 had significantly fewer species than groups 1 and 2 (p <0.05). The number of individuals was variable among groups, with group 1 having the lowest number of individuals (Table 2). However, there was no significant difference among groups in the mean number of individuals per sampling site (ANOVA ONE-WAY, F_[2, 30]_ = 1.43, p = 0.25). The overall diversity of the metazoan parasite component communities of *S. gunteri* is presented in Table 2. There were significant differences among groups in Simpson’s diversity index (ANOVA ONE-WAY, F_[2, 30]_ = 5.50, p = 0.009), with Tukey’s test indicating that group 3 had a significantly lower Simpson’s diversity index than groups 1 and 2 (p <0.05). There were also significant differences among groups in the numerical dominance expressed by the Berger-Parker dominance index (ANOVA ONE-WAY, F_[2, 30]_ = 5.97, p = 0.006), with Tukey’s test indicating that group 3 had a significantly higher numerical dominance than groups 1 and 2 (p <0.05).

**Table 2:** **The component communities of the metazoan parasites of the shoal flounder** ***Syacium gunteri***

**Metrics**	**Overall**	**Group 1**	**Group 2**	**Group 3**
Total number of species	13	12	10	4
Total number of individuals	8760	1762	4605	2393
Mean number of individuals within sampling sites	265.45 ± 221.10	328.92 ± 260.74	176.20 ± 173.14	265.89 ± 185.27
Simpson’s Diversity index	0.29 ± 0.25	0.29 ± 0.21	0.45 ± 0.30	0.10 ± 0.10
Berger-Parker dominance index	0.82	0.59	0.84	0.94
Numerically dominant species	*O. wageneri*	*O. wageneri*	*O. wageneri*	*O. wageneri*
Colonization strategy	Autogenic	Autogenic	Autogenic	Autogenic
Specialist/generalist status	Generalist	Generalist	Generalist	Generalist
Similarity within sampling sites (Jaccard)	0.43 ± 0.23	0.58 ± 0.19	0.21 ± 0.22	0.81 ± 0.17
Similarity within sampling sites (percentage of similarity)	36.43 ± 27.74	38.91 ± 24.92	19.07 ± 23.22	53.72 ± 24.32

The data include the community metrics for 33 sampling sites (Overall) and for the three groups of sampling sites 1 to 3 formed by the cluster analysis (Figure 2) in the Campeche Sound, southern Gulf of Mexico. *O. wageneri* is *Oncomegas wageneri*.

The qualitative similarity of the component community within the groups was variable, with group 3 having the highest similarity (0.81 ± 0.17) within its sampling sites (Table 2). There were significant differences among groups in Jaccard’s index of qualitative similarity (ANOVA ONE-WAY, F_[2, 169]_ = 97.36, p <0.001), with Tukey’s test indicating that group 3 had a significantly higher qualitative similarity than groups 1 and 2 (p <0.05). The quantitative similarity of the component communities within groups was also variable, with group 3 having the highest similarity (53.72 ± 24.32) within its sampling sites (Table 2). There were also significant differences among groups in the percentage of similarity index of the component communities (ANOVA ONE-WAY, F_[2, 169]_ = 20.93, p <0.001), with Tukey’s test indicating that group 3 had significantly higher quantitative similarity than groups 1 and 2 (p <0.05).

### Infracommunities

The metazoan parasite infracommunities of the shoal flounder for the overall group and groups 1 through 3 are presented in Table 3, with group 3 having the lowest mean number of species. There were significant differences in the mean number of species per fish among groups (ANOVA ONE-WAY, F_[2, 200]_ = 18.40, p <0.001), with Tukey’s test indicating that group 3 had significantly fewer species than groups 1 and 2 (p <0.05). The mean number of individual parasites per fish among groups was variable. However, no significant difference among groups in the mean number of individuals per fish was detected (ANOVA ONE-WAY, F_[2, 200]_ = 0.17, p = 0.85). The overall values of the Brillouin’s diversity index for the parasite infracommunities of *S. gunteri* can be seen in Table 3. There were significant differences in the mean Brillouin’s diversity index among groups (ANOVA ONE-WAY, F_[2, 200]_ = 305.69, p <0.001), with Tukey’s test indicating that group 3 had significantly lower values of the diversity index than groups 1 and 2 (p <0.05). There were also significant differences among groups in the numerical dominance expressed by the mean Berger-Parker dominance index per individual fish (Kruskal-Wallis test, H = 17.28, p <0.001, n = 203), with the rank test indicating that group 2 had significantly lower numerical dominance than groups 1 and 3 (p <0.05).

**Table 3:** **The infracommunities of the metazoan parasites of the shoal flounder** ***Syacium gunteri***

**Community metrics**	**Overall**	**Group 1**	**Group 2**	**Group 3**
Mean number of species	1.89 ± 1.02	1.86 ± 1.88	2.58 ± 1.26	1.42 ± 0.76
Mean number of individuals	43.71 ± 45.79	44.98 ± 50.17	40.97 ± 40.22	43.51 ± 41.83
Brillouin’s diversity index	1.16 ± 0.53	1.42 ± 0.22	1.53 ± 0.25	0.38 ± 0.23
Berger-Parker dominance index	0.84 ± 0.23	0.86 ± 0.19	0.73 ± 0.21	0.87 ± 0.29
Numerically dominant species	*O. wageneri*	*O. wageneri*	*O. wageneri*	*O. wageneri*
Mean similarity within sampling sites (Jaccard)	0.78 ± 0.13	0.79 ± 0.13	0.69 ± 0.13	0.86 ± 0.10
Mean similarity within sampling sites (percentage of similarity)	60.36 ± 17.66	59.91 ± 17.73	56.22 ± 16.06	66.89 ± 17.17

The data included the community metrics for 33 sampling sites (overall) and for the groups of sampling sites 1 to 3 formed by the cluster analysis (Figure 2) in the Campeche Sound, southern Gulf of Mexico. *O. wageneri* is *Oncomegas wageneri*.

The mean qualitative similarity of the infracommunity within groups was variable, with group 3 having the highest similarity (0.86 ± 0.10) within its sampling sites (Table 3). There were significant differences among groups in Jaccard’s index of qualitative similarity (ANOVA ONE-WAY, F_[2, 7194]_ = 546.56, p <0.001, n = 7197 pair comparisons), with Tukey’s test indicating that group 3 had significantly higher qualitative similarity than groups 1 and 2 (p <0.05). The quantitative similarity of the infracommunity within groups was also variable, with group 3 having the highest similarity (66.89 ± 17.17) within its sampling sites (Table 3). There were significant differences among groups in the quantitative similarity of the infracommunity (ANOVA ONE-WAY, F_[2, 7536]_ = 128.61, p <0.001), with Tukey’s test indicating that group 3 had a significantly higher numerical dominance than groups 1 and 2 (p <0.05).

### Qualitative and quantitative similarity among the groups of sampling sites

The qualitative and quantitative similarities between pairs of groups of sampling sites are presented in Table 4. The highest qualitative and quantitative similarities at the component community level were between groups 1 and 3 (qualitative: 0.64 ± 0.21; quantitative: 43.11 ± 25.55). However, there were significant differences among the groups in terms of qualitative similarity (ANOVA ONE-WAY, F_[2, 169]_ = 97.36, p <0.001, n = 172), with Tukey’s test indicating significant differences among the three groups of sampling sites (p <0.05). For the quantitative similarity at the component community level, there were significant differences among the groups (ANOVA ONE-WAY, F_[2, 169]_ = 20.93, p <0.001, n = 172), with Tukey’s test indicating significant differences among the three groups of sampling sites (p <0.05).

**Table 4:** **Similarity of the metazoan parasite communities of the shoal flounder** ***Syacium gunteri***

	**Group 1**	**Group 2**	**Group 3**
Group 1	X	C 0.45 ± 0.27	C 0.64 ± 0.21
		I 0.78 ± 0.13	I 0.81 ± 0.12
Group 2	C 32.34 ± 26.03	X	C 0.47 ± 0.36
	I 59.36 ± 17.53		I 0.80 ± 0.14
Group 3	C 43.11 ± 25.55	C 34.47 ± 29.24	X
	I 61.47 ± 17.84	I 62.85 ± 17.54	

The mean values of the qualitative and quantitative similarity (± standard deviation) among groups of sampling sites are presented at component (C) and infracommunity (I) levels. Data in the upper triangle are Jaccard’s similarity index values. Data in the lower triangle are values of the percentage of similarity index.

At the infracommunity level, there were significant differences in the qualitative similarity among the groups (ANOVA ONE-WAY, F_[2, 7194]_ = 546.56, p <0.001), with Tukey’s test indicating that group 3 had significantly higher similarity values than groups 1 and 2 (p <0.05). For the quantitative similarity, there were also significant differences among the groups (ANOVA ONE-WAY, F_[2, 7536]_ = 128.61, p <0.001), with Tukey’s test indicating that group 3 had significantly higher similarity values than groups 1 and 2 (p <0.05).

### Statistical associations between environmental and biological variables

**Figure 3:**
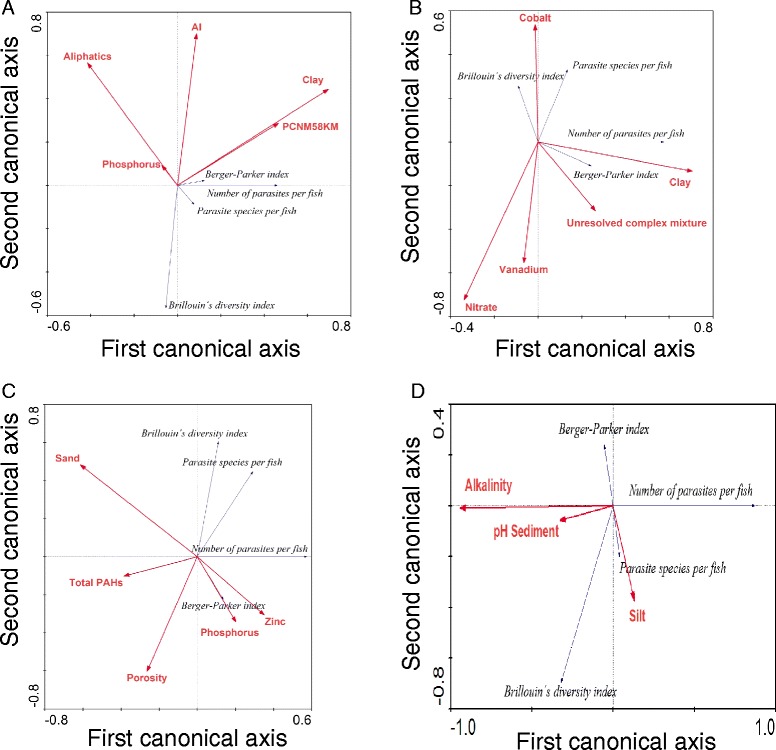
**A-D Redundancy analysis of the environmental variables and infracommunity metrics of the parasites of** ***Syacium gunteri*** **. (A)** The RDA for all sampling sites accounted for 36% of the total variance, and was highly significant for the first and all four canonical axes (F = 51.46; P-value = 0.0002; 4999 permutations) for the 33 sampling stations. **(B)** For group 1, the RDA accounted for 35% of the total variance, and was highly significant for the first and all four canonical axes (F = 51.46; P-value = 0.0002; 4999 permutations). **(C)** For group 2, the RDA accounted for 23% of the total variance, and was highly significant for the first and all four canonical axes (F = 14.10; P-value = 0.03; 4999 permutations). **(D)** For group 3, the RDA accounted for 68% of the total variance, and was highly significant for the first and all four canonical axes (F = 38.24; P-value = 0.03; 4999 permutations).

Table 5 shows the environmental variables and contaminants in the sediment and water selected by the forward stepwise procedure of CANOCO for all 33 sampling sites and for each of the three groups of sampling sites in Figure 2. These variables explained the greatest percentage of the variance in the redundancy analysis (Figure 3A-D). Figure 3A shows the statistical associations between the five environmental variables explaining the greatest amount of variance and the parasite infracommunity metrics. The most relevant pattern in Figure 3A was the negative association between the infracommunity metrics (especially Brillouin’s diversity index and the number of parasites per fish) and the concentrations of aliphatic hydrocarbons, aluminium and phosphorus. In contrast, the number of parasites per fish had a positive association with the concentration of clay in the sediments and an unknown environmental variable acting at a spatial scale of 58 km (PCNM58KM). Figure 3B-D show the statistical associations between the environmental variables explaining the greatest amount of variance and the infracommunity metrics for groups 1, 2 and 3, respectively. Figure 3B shows the negative statistical associations of nitrate, vanadium and the unresolved complex mixture with Brillouin’s diversity index and the number of parasite species per fish. For these two community metrics, the concentration of cobalt was apparently irrelevant. The number of parasites per fish and the Berger Parker dominance index were both associated positively with clay and negatively with nitrate and vanadium. Figure 3C shows a negative statistical association between total PAHs, porosity and phosphorus and most of the infracommunity metrics. The exception was the Berger-Parker dominance index, which apparently increased with the concentration of phosphorus. Figure 3D shows that for group 3, the number of individuals per fish had a negative association with the alkalinity and pH of the sediment. Additionally, with an increasing silt concentration in the sediments, the number of parasite species per fish and the Brillouin’s diversity index increased, and Berger-Parker index values decreased.

**Table 5:** **Selected environmental variables from the sampling sites in the Gulf of Mexico**

	**All sampling sites**		**Group 1**		**Group 2**		**Group 3**	
**Physicochemical variables**	**Mean ± SD**	**Range**	**Mean ± SD**	**Range**	**Mean ± SD**	**Range**	**Mean ± SD**	**Range**
Alkalinity (meq.L^-1^)	2.64 ± 0.41	2.92-0.84	2.72 ± 0.13	2.55-2.92	2.60 ± 0.59	0.84-2.92	**2.55 ± 0.50**	**1.44-2.92**
Clay (%)	**24.41 ± 15.10**	**0-55.00**	**24.53 ± 1.00**	**0- 55.00**	28.00 ± 0.00	0- 55.00	18.49 ± 0.00	0-30.00
Silt (%)	15.00 ± 9.92	0-30.00	13.40 ± 8.87	2.00-30.00	17.73 ± 10.51	0- 30.00	**14.14 ± 11.68**	**0.00-30.00**
PCNM58K	**−3.04 ± 21.20**	**−62.31-21.62**	−8.36 ± 20.47	−39.63-21.62	−0.45 ± 25.65	−62.31-21.62	4.31 ± 13.33	−12.28-21.62
pH sediments	7.73 ± 0.21	7.45-8.33	7.61 ± 0.13	7.45-8.33	7.82 ± 0.26	7.59-8.33	**7.84 ± 0.16**	**7.72-8.17**
Porosity (%)	0.51 ± 0.08	0.26-0.64	0.53 ± 0.07	0.34-0.64	**0.50 ± 0.10**	**0.26-0.60**	0.49 ± 0.09	0.33-0.60
Sand (%)	60.59 ± 21.55	20.00-100	62.07 ± 19.34	20.00-97.00	**54.27 ± 25.65**	**20.00-100**	67.37 ± 19.40	47.00-100.00
**Heavy metals**								
Aluminum (μg.g^−1^)	**4.75 ± 4.14**	**0.24-18.52**	2.83 ± 3.15	0.24-12.15	7.68 ± 5.00	1.00-18.52	4.25 ± 1.19	1.78-5.76
Cobalt (μg.g^−1^)	0.23 ± 0.07	0.05-0.49	**0.24 ± 0.10**	**0.05-0.49**	0.21 ± 0.07	0.05-0.31	0.24 ± 0.01	0.23-0.26
Vanadium (μg.g^−1^)	0.59 ± 0.87	0.02-4.01	**1.17 ± 1.05**	**0.10-4.01**	0.10 ± 0.05	0.02-0.20	0.13 ± 0.07	0.04-0.28
Zinc (μg.g^−1^)	0.88 ± 0.54	0-1.86	0.44 ± 0.34	0.00-1.14	**1.09 ± 0.36**	**0.49-1.86**	1.52 ± 0.20	1.11-1.78
**Hydrocarbons**								
Aliphatics (μg.g^−1^)	**0.30 ± 0.32**	**0-1.28**	0.18 ± 0.00	0-0.45	0.45 ± 0.00	0-1.28	0.31 ± 0.00	0-0.74
Total PAH (μg.g^−1^)	1.31 ± 0.51	0.48-2.37	1.29 ± 0.52	0.48-2.37	**1.38 ± 0.58**	**0.69-2.15**	1.24 ± 0.44	0.79-2.09
UCM (μg.g^−1^)	11.65 ± 8.66	0.72-30.98	**12.24 ± 8.41**	**1.45-26.79**	13.96 ± 10.04	0.72-30.98	6.74 ± 5.27	0.91-16.23
**Nutrients**								
Nitrates (μM)	1.90 ± 3.04	0.06-15.74	**2.75 ± 4.28**	**0.20-15.74**	1.25 ± 1.09	0.06-2.69	1.10 ± 1.06	0.20-2.69
Phosphorus (μM)	**5.37 ± 1.22**	**1.85-6.50**	5.27 ± 1.29	2.30-6.50	**5.42 ± 1.32**	**1.85-6.38**	5.53 ± 1.06	3.38-6.38

The variables in bold are the ones that explained the highest percentage of variance in the redundancy analysis for both the overall analysis (33 sampling sites) and for each one of the groups of sampling sites in Figure 2. The PCNM58K corresponds to a spatial variable acting at the spatial scale of 58 km. Total PAH = total polycyclic aromatic hydrocarbons, UCM = unresolved complex mixture.

## Discussion

The results show that there were significant statistical associations between environmental variables and contaminants and the parasite community metrics of the shoal flounder. Therefore, the environmental impacts primarily produced by river run-off and polyaromatic hydrocarbons in the study area apparently have a direct effect on the parasite community metrics of *S. gunteri*. The sampling sites in groups 1 and 2 had a relatively high number of species, high diversity values and low qualitative and quantitative similarity at the component and infracommunity levels (Tables 2 and 3). Thus, despite the presence of pollutants (Table 5), the environmental conditions in both groups of sites were apparently not bad enough to prevent the life cycles from being completed. Consequently, we considered these groups of sampling sites as mildly disturbed. In contrast, as detailed below, several of the component and infracommunity metrics of the sites of group 3 presented values suggesting alterations related to environmental impact, including a low number of species, high numerical dominance and high similarity within sites (Tables 2 and 3). These characteristics suggest that parasite community metrics, and hence the probability of completion of the life cycles, may have been affected. Consequently, we considered the sampling sites in group 3 as strongly disturbed.

### Prevalence and abundance of the most frequent and abundant species

All of the metazoan parasites recovered were generalist autogenic parasites that have been reported in marine and brackish fishes from other families in the region, namely, the red grouper *Epinephelus morio* [33], the grey snapper *Lutjanus griseus* [34], the tonguefish *Symphurus plagiusa* [35], the Florida pompano *Trachinotus carolinus* [36], the Mayan sea catfish *Ariopsis assimilis* [13] and the Mexican flounder *Cyclopsetta chittendeni* [37].

The most frequent and abundant helminth species infecting the shoal flounder across all 33 sampling sites were the larval cestode *O. wageneri* and the larval nematode *P. decipiens* (Table 1). The most likely explanation for the presence of these larval species throughout the study area is that they use copepods, amphipods, shrimp, and isopods as intermediate hosts, which in turn form part of the diet of *S. gunteri* [38-41]. In the case of *P. decipiens*, the adults of this nematode have been reported in pygmy whale (*Kogia breviceps*) along the coast of the Yucatan Peninsula [42]. In the case of *O. wageneri*, the putative definitive hosts are the stingrays, *Dasyatis centroura* [40].

Table 1 shows the low number of metazoan parasite species found at the sites of group 3, where only 4 species were recovered, versus sites in groups 1 and 2, where 10 and 12 parasite species were present, respectively. The most likely explanation for this difference is the absence of suitable conditions for the completion of the life cycle of all other parasite species in the sites of group 3. This is evidently the case for adult helminths such as *Lecithochirium* sp., the nematode Capillariidae gen. sp., the acanthocephalan *Serrasentis sagittifer*, the larval digenean *Stephanostomum* sp. and the copepod *Caligus pomacentrus*. These results suggest that a number of environmental characteristics prevent the presence of intermediate hosts in the sites of group 3. Similar effects of anthropogenic environmental impacts on the parasite populations of flatfishes have been reported for European waters [43,44] and for the Mexican flounder *Cyclopsetta chittendeni* from the Campeche Sound, Gulf of Mexico [37].

### Component communities

The parasite species composition of the component communities of the shoal flounder was formed by a large number of species in the larval stage (8/13 = 61%), which is most likely because this fish species is a benthic predator feeding on shrimps and copepods [41]. The shoal flounder had a lower number of parasite species compared with *C. chittendeni* (17 species; [37]) but a higher number compared with *S. plagiusa* in the Gulf of Mexico (8; [35]). In comparison with flatfishes from other regions, the number of parasite species of the shoal flounder was in the range reported for Spain and Germany for *Platichthys flesus* (13 and 17 species, respectively) [45,46]. However, there were fewer parasite species in *S. gunteri* than in *Hippoglossina macrops* and *Paralichthys adspersus* from the coasts of Brazil and Chile, which harbour 20 and 22 helminth species, respectively [47,48].

The overall diversity of the parasite component communities of *S. gunteri* (Table 2) is similar to that of *S. plagiusa* (0.24 ± 0.11) reported in the Gulf of Mexico [35]. However, the diversity of the component community for the sites of group 3 is the lowest obtained in the region to date for any flatfish species (see Table six in [37]). The statistically significant low values of diversity, the mean number of metazoan parasite species per site and the significantly high values of numerical dominance and similarity for the sites of group 3 (Table 2) were most likely due to the large number of larval parasites and the unsuitable conditions for the completion of the life cycle of the parasites using intermediate hosts. Further support for this argument comes from the study of benthic bioindicators, in which benthic fauna affected by oil spills or organic matter deposition undergo changes in their community composition from contaminant-sensitive species to contaminant-resistant species [49,50]. Because the intermediate hosts (e.g., mollusks, shrimps) of the metazoan parasites are also residents of the benthic zone, their removal could negatively affect the parasite life cycles at the sites of group 3. In contrast, the completion of the life cycle of all other parasites in Table 1 was apparently not affected at the sites of the less environmentally disturbed groups 1 and 2. With increasing levels of environmental contamination, similar patterns of decreasing species numbers and parasite community metrics in flounder (*Platichthys flesus*) and of the infection parameters of digeneans in Mayan sea catfish (*Ariopsis assimilis*) have been reported by Schmidt et al. [46] and Vidal-Martínez et al. [13] , respectively.

### Infracommunities

The mean number of metazoan parasite species per fish of the shoal flounder (Table 3) was within the range previously described for the Mexican flounder *C. chittendeni* (1.19 ± 1.17 to 3.27 ± 1.64 species per fish; [37]) and for the tonguefish *S. plagiusa* (2.00 ± 0.55 to 3.00 ± 0.66; [35]). These results suggest that the shoal flounder individuals are acquiring similar numbers of metazoan parasite species compared to *C. chittendeni* and *S. plagiusa* in the Gulf of Mexico, which is not surprising since these flatfish species share the same regional pool of parasites. However, once again, sites of group 3 had the fewest species per fish in the present study (Table 3). The mean number of individual parasites per fish (Table 3) in the shoal flounder was also within the range found for *S. plagiusa* in the Campeche Sound (47.00 ± 31.81) [35]. However, the identity of the numerically dominant species was different, with *O. wageneri* for *S. gunteri* and the larval digenean *Stephanostomum* sp. for *S. plagiusa*. This difference in the identity of the numerically dominant species reflects the oceanic versus coastal character of the parasite fauna of the shoal flounder and the tonguefish, respectively.

The diversity values for the parasite infracommunities of *S. gunteri* fall within the range commonly observed in the Campeche Sound, but the diversity values in sites of group 3 were the lowest obtained in the present study (see [35], and Table six in [37]). The statistically significant low diversity and mean numbers of metazoan parasite species per fish and the significantly high values of numerical dominance and similarity of sites of group 3 (Table 3) were most likely due to the large numbers of larval parasites and the unsuitable conditions for the parasites’ life cycles. Similar numerical dominance and high similarity values at the infracommunity level were observed in the parasite communities of the Mexican flounder *C. chittendeni* [37].

The coincidence in the numerical dominance of larval parasites of the infracommunities of the three flatfish species studied in the Campeche Sound, namely, the larval cestode *O. wageneri* in *S. gunteri,* the larval nematode Ascarididae gen. sp. *in C. chittendeni* and the larval digenean *Stephanostomum* sp. in *S. plagiusa*, is remarkable. Whether this pattern reflects a transition in the parasite community composition of the three flatfishes from contaminant-sensitive parasite species to contaminant-resistant parasite species is difficult to assess. However, because the three flatfish species were captured in the Campeche Sound, a zone influenced by oil extraction activities and the discharge of the Grijalva-Usumacinta River, the community metrics of their metazoan parasite fauna likely reflect the effect of living in a highly disturbed environment. To undertake such a comparison, the parasite communities of flatfishes in other, less-impacted environments in the Gulf of Mexico must be assessed.

### Qualitative and quantitative similarity among groups at the component and infracommunity levels

At the component community level, the highest qualitative and quantitative similarities were observed between sites of groups 1 and 3 (Table 4), although the similarity values were rather low (Table 4). The most likely explanation for these similarities is that there were only a few parasite species that were shared between the sites. Clearly, this is the case for the sites of groups 1 and 3 since there were more parasite species in the former than in the latter (Table 1). These low similarities at the component community level are typical of diverse parasite communities. For example, flatfish species such as *Pleuronectes flesus*, *Hippoglossoides platessoides*, *Gyptocephalus cynoglossoides* and *Hippoglossus hippoglossus* had quantitative similarities between 24 ± 25 to 53 ± 19 for intestinal parasite communities in Norway [43,51].

At the infracommunity level, the qualitative and quantitative similarities were very high among all three groups of sampling sites, especially for Jaccard’s similarity index (Table 4). The most likely explanation for this pattern is that at this hierarchical level, the individual fish were sharing the most frequent and abundant species of larval parasites (Table 1). This pattern can be explained based on the concept of “seed rain” [52], in which the larvae of the parasites become dispersed by ocean or tidal coastal currents, infecting intermediate hosts, which are eventually eaten by the shoal flounder at all of the study sites. This larval dispersion process has been well documented for free-living stages of digeneans (cercariae) infecting benthic intermediate hosts (crabs) [52-54].

### Statistical associations between environmental variables and biological variables

The positive statistical associations in Figure 3A between both the number of parasites per fish and the Berger-Parker dominance index of the 33 sampling sites and the concentrations of aliphatic hydrocarbons, aluminium and phosphorus suggest a positive effect of these chemicals on these infracommunity metrics. The concentrations of aliphatic hydrocarbons (range 0–1.28 μg.g^−1^) and aluminium (range 0.24-18.52 μg.g^−1^; Table 5) found in the present study did not exceed the normal average concentrations in deep-sea sediments (10 μg.g^−1^ for aliphatics [55]; 95 μg.g^−1^ for aluminium [56]). A potential explanation for these associations is that the presence of aliphatic hydrocarbons and phosphorus, transported from the continent through rivers and then discharged into the marine sediments, enhances the growth of hydrocarbonoclastic bacteria [57]. The increase in these bacteria would in turn enhance primary and secondary productivity in the area, with a consequent increase in the number of intermediate hosts, as has been suggested for regions affected by oil spills such as the Prestige [8,9]. The Brillouin’s diversity index had a negative statistical association with all of the environmental variables in Figure 3A. Whether some of these parasite species are sensitive to contamination remains an open question, however the combination of the environmental variables in Figure 3A is apparently having negative effects on this parasite community metric. The number of parasites per fish also had a positive association with the concentration of clay in the sediments and other unknown environmental variables acting at a spatial scale of 58 km (PCNM58KM), which could presumably be ocean currents transporting infective stages. Thus, an environment with a low concentration of aliphatic hydrocarbons, aluminium and clay seems to allow the intermediate hosts to survive, which in turn enhances transmission within these parasite communities at a spatial scale of 58 km.

The negative statistical associations in group 1 (Figure 3B) between nitrate, vanadium and the unresolved complex mixture, and the values of the Brillouin’s diversity index and the numbers of parasites and parasite species per fish suggest a deleterious effect of these chemicals on the infracommunity metrics. The nitrate concentrations in sites of group 1 (Figure 3B) were between 0.06 and 15.76 μM (Table 5), and concentrations of this nutrient greater than 0.59 μM are generally considered harmful for aquatic organisms [58]. This value was exceeded at 8 out of 14 sampling sites in group 1 (data not shown). The most likely negative effect would be on intermediate hosts such as mollusks or benthic crustaceans. The problem is apparently not extreme at present because the parasite community still has a relatively large number of species and values of diversity similar to those of parasite communities in the region (see Table six in [37]). However, if this uncontrolled sewage discharge continues, hypoxia similar to that caused by the nutrients deposited in the Mississippi river [59] can be expected. For the sites in group 1, the concentrations of vanadium, the unresolved complex mixture (UCM) and cobalt were between 0.02 and 4.0 μg.g^−1^, 0.72 and 30.98 μg.g^−1^ and 0.05 and 0.49 μg.g^−1^, respectively (Table 5). None of these concentrations exceeded the sediment benchmarks for aquatic life proposed for vanadium (57 μg.g^−1^ [60]), UCM (see Table one in [61]), or cobalt (50 mg.g^−1^ for aquatic organisms) by the EPA [62]. The percentage of clay in the sediments (0-55%) is apparently adequate for the completion of parasite life cycles.

In the sites of group 2 (Figure 3C), there were negative statistical associations between porosity and total concentrations of hydrocarbons (total PAHs), phosphorus and zinc and all of the parasite infracommunity metrics (with the exception of the Berger Parker dominance index). The increase in porosity could theoretically have a positive effect on the probability of the presence of invertebrates [63]. If this is the case, predators of the potential intermediate hosts could reduce the probability of parasite transmission. In addition, the total PAH concentrations for the sites of group 2 ranged from 0.68 to 2.15 μg.g^−1^ (Table 5), whereas the threshold effect level (TEL) of total PAHs reported by NOAA (National Oceanic and Atmospheric Administration) is 1.68 μg.g^−1^ for marine organisms. In group 2, 4 out of 10 sampling sites (40%) slightly exceeded this threshold. The phosphorus concentrations ranged from 1.09 to 6.50 μM, and the zinc concentrations ranged from 0 to 1.86 μg^−1^ (Table 5). These values were low; the Canadian Water Quality Guidance states that water with 129.15 μM of phosphorus is oligotrophic [64]. In the same way, the zinc concentrations were below the threshold effect level for zinc in sediments (123 μg.g^−1^; [65]). However, a possible synergistic effect, especially with porosity and total PAHs, cannot be ruled out because higher porosity indicates more interstitial space for PAHs, phosphorus and zinc. Our results are in accordance with those of Vidal-Martínez et al. [14], who found negative correlations between the number of symbionts in the gills of pink shrimp (*Penaeus duorarum*) and the UCM concentrations in sediments from the Campeche Sound. UCM contains both aliphatic and polyaromatic hydrocarbons, which in turn are related to the total PAHs present in the sediments [55].

The results for the sites of group 3 (Figure 3D) suggest that there was a decrease in the number of individual parasites per fish with decreasing alkalinity and silt proportion. The range of alkalinity was between 0.84 and 2.92 meq.L^-1^ (Table 5), within the normal range for the Gulf of Mexico (2.46-2.54 meq.L^-1^; [66]) and other saline-alkaline environments (2.5 meq.L^-1^; [67]). The proportion of silt was also within the range found in the southern Gulf of Mexico [68]. Therefore, the alkalinity and the silt proportions for the study area do not seem to pose any threat to the parasites. Other community metrics in Figure 3D, for example the parasite species per fish, increased with increasing silt proportion. Brillouin’s diversity index also increased with the alkalinity values. The pH range in the sediments was small, between 7.45 and 8.33 (Table 5). However, our results suggest that there were very few parasites with complex life cycles in the sites of group 3 (Table 1). Thus, apparently this narrow range of pH could be enough to create an inhospitable environment for parasites with complex life cycles. In further support of this hypothesis, pH values between 7.7 and 7.9 increased the mortality of larval brittlestar *Ophiothrix fragilis* in comparison with the control pH value of 8.1 [69]. For parasites, Koprivnikar et al. [70] reported an interaction between pH (range = 7.8 to 8.2), salinity and time affecting the survival of the larval stages of the digenean *Acanthoparyphium spinulosum.* Thus, the potential interaction between pH and the decrease in the number of parasites cannot be disregarded without experimental evidence.

An equally possible explanation is that there were other contaminants that were not included in the present study and that could be acting alone or synergistically to negatively affect the parasite community metrics of group 3. This unknown contaminant could be co-varying strongly with both alkalinity and pH in the sediment, which could be the reason that these two variables appear significant in the analysis. Potential candidates for this unknown contaminant are PCBs and pesticides, as negative statistical associations have been found between these contaminants and the number of gill symbionts of pink shrimp in the Campeche Sound [14].

## Conclusions

Our results suggest that different combinations of environmental and contaminant variables affect the three groups of sites detected and that this influence is spatially highly patchy, acting at different spatial scales. This finding poses a very complex scenario in which parasites could be responding specifically to different combinations of contaminants. Most of the environmental variables did not exceed the limits established by different agencies (EPA, NOAA), with the exception of nitrate and total PAHs, which were above the values established in the literature, although the number of sites above the threshold was low in most cases. Thus, both the shoal flatfish and its parasites apparently live in tolerable levels of these hydrocarbons and nutrients in most cases. In the specific case of nitrate, if this uncontrolled sewage discharge continues, the occurrence of hypoxia similar to that caused by the Mississippi river [59] can be expected. In examining sites of group 3 specifically, other contaminants such as PCBs and pesticides should be included to obtain a more complete picture of the potential contaminants affecting the parasite community metrics. The unexpected combined effect of alkalinity and pH upon the parasites is very suggestive, and we speculate that the potential interaction between pH and the decrease in the number of parasites cannot be disregarded. The community metrics chosen generally had robust statistically significant associations with both physicochemical and contaminant variables, which supports the ecological relevance of these parameters as indicators of aquatic environmental health [5,71].

## Additional file

Additional file 1:
**Environmental variables of sediments and water from the sampling sites in the Gulf of Mexico.** Codes are as follows: BPM/APM = ratio of PAHBPM and PAHAPM, PAHAPM = polycyclic aromatic hydrocarbons of high molecular weight (4–5 benzene rings), PAHBPM = polycyclic aromatic hydrocarbons of low molecular weight (2–3 benzene rings), PAHTOT = total polycyclic aromatic hydrocarbons, PCNM2, PCNM15, PCNM 21 and PCNM58K correspond to spatial variables acting at the spatial scales of 2, 15, 21 and 58 km respectively, UCM = unresolved complex mixture.
